# Risk Assessment of Toxic and Potentially Toxic Metals in Raw Goat Milk: A Systematic Review of Global Data and Environmental Factors

**DOI:** 10.1111/1541-4337.70268

**Published:** 2025-09-01

**Authors:** Arlen Carvalho de Oliveira Almeida, Paloma Almeida Rodrigues, Marion Pereira da Costa, Carlos Adam Conte‐Junior

**Affiliations:** ^1^ Center for Food Analysis (NAL), Technological Development Support Laboratory (LADETEC) Federal University of Rio de Janeiro (UFRJ) Rio de Janeiro Rio de Janeiro Brazil; ^2^ Laboratory of Advanced Analysis in Biochemistry and Molecular Biology (LAABBM), Department of Biochemistry Federal University of Rio de Janeiro (UFRJ) Rio de Janeiro Rio de Janeiro Brazil; ^3^ Analytical and Molecular Laboratorial Center (CLAn), Institute of Chemistry (IQ) Federal University of Rio de Janeiro (UFRJ) Rio de Janeiro Rio de Janeiro Brazil; ^4^ Graduate Program in Food Science (PPGCAL), Institute of Chemistry (IQ) Federal University of Rio de Janeiro (UFRJ) Rio de Janeiro Rio de Janeiro Brazil; ^5^ Laboratory of Technology and Inspection of Milk and Derivatives (LaITLácteos), School of Veterinary Medicine Federal University of Bahia (UFBA) Salvador Bahia Brazil; ^6^ Graduate Program in Veterinary Hygiene (PPGHV), Faculty of Veterinary Medicine Fluminense Federal University (UFF) Niterói Rio de Janeiro Brazil; ^7^ Graduate Program in Chemistry (PGQu), Institute of Chemistry (IQ) Federal University of Rio de Janeiro (UFRJ) Rio de Janeiro Rio de Janeiro Brazil

**Keywords:** bioaccumulation, caprine, dairy products, environmental contamination, environmental factors, food safety, heavy metals, systematic review

## Abstract

Goat milk is a culturally and nutritionally significant food worldwide, yet its safety regarding toxic and trace elements remains poorly defined. The absence of geographically balanced surveillance and the variability in contamination across rural, urban, and industrial environments limit reliable risk assessment for human exposure. We hypothesized that raw goat milk acts as a sentinel matrix for chronic exposure to toxic and potentially toxic elements, with risks strongly influenced by production systems and environmental contexts. Accordingly, the objective of this review was to systematically synthesize evidence from 20 studies conducted across Asia, Europe, and North Africa, quantifying toxic metals (arsenic, lead, cadmium, and mercury) and essential elements (chromium, nickel, copper, zinc, iron, manganese, and aluminum) relative to thresholds established by the World Health Organization (WHO), the European Food Safety Authority (EFSA), and the Institute of Medicine (IOM). Results revealed pronounced heterogeneity, with lead reaching 0.382 ± 0.0026 mg/L in rural Algeria, chromium peaking at 16.423 ± 0.349 mg/L in industrial Asaluyeh and 14.211 ± 0.205 mg/L in rural Kaki, Iran, and pediatric estimated daily intakes (EDIs) for Cr up to 2.74 × 10^−1^ mg/L body weight/day, whereas target hazard quotients (THQs) and hazard index (HI) values exceeded safety thresholds by up to two orders of magnitude. These findings demonstrate that raw goat milk is a heterogeneous yet consistent vector of chronic exposure to toxic elements, demanding harmonized monitoring frameworks, regionally adapted regulatory standards, and integrated risk assessments coupling chemical surveillance with agroecological and public health strategies.

## 1 Introduction

Goat milk represents a critical nutritional resource for millions of individuals worldwide, particularly in rural and semi‐arid regions where it frequently serves as a primary source of protein, calcium, and bioavailable micronutrients. Its biochemical composition, characterized by high digestibility and a favorable fatty acid its biochemical composition, including a favorable fatty acid profile, characterized by higher proportions of medium‐chain triglycerides and bioavailable micronutrients, supports its nutritional value, supports its widespread inclusion in dietary strategies for children, older adults, and individuals with cow milk allergy (Shkembi and Huppertz 2021). However, despite its recognized nutritional and cultural importance, goat milk may also function as a vector for human exposure to toxic and potentially toxic metals arising from environmental contamination (Homayonibezi et al. 2021).

The accumulation of metals in milk is a multifactorial process driven by a continuum of geochemical, ecological, and physiological determinants. Soil mineralogy, forage composition, water quality, and atmospheric deposition collectively govern the bioavailability of elements to grazing animals (Meena et al. 2020). Geochemical properties such as pH, cation exchange capacity, and the metal‐binding potential of soil–plant systems determine the extent to which metals are transferred into animal diets. Once ingested, absorption and partitioning within animal tissues and fluids are influenced by solubility, competition with essential elements, and the animal's metabolic and physiological state, leading to variable secretion into milk (Miclean et al. 2019).

Industrial emissions, mining, and the widespread application of phosphate fertilizers amplify these dynamics, enabling the entry of hazardous metals, including lead (Pb), cadmium (Cd), arsenic (As), and mercury (Hg), into the food chain, with pronounced implications for public health (Rajeswari et al. 2024). Arsenic and cadmium are classified as Group 1 carcinogens by the International Agency for Research on Cancer (IARC) (EFSA 2024), chronic lead exposure is linked to irreversible neurodevelopmental deficits in children (WHO 2023), and methylmercury is associated with cognitive impairment during early developmental stages (Rodrigues et al. 2024).

Although trace elements such as iron (Fe), copper (Cu), zinc (Zn), manganese (Mn), chromium (Cr), and nickel (Ni) are indispensable for maintaining metabolic homeostasis, concentrations exceeding physiological thresholds can exert deleterious effects (EFSA 2024). For example, manganese, essential for enzymatic regulation, has been implicated in neurotoxicity when excessively accumulated within the central nervous system (Liu et al. 2020). Likewise, elevated nickel intake has been associated with hypersensitivity reactions and systemic toxicities (EFSA 2020). The duality between nutritional adequacy and toxic overload highlights the necessity of rigorously quantifying both toxic and essential elements in goat milk within environmentally diverse contexts.

Accordingly, this systematic review aims to (i) synthesize and critically analyze the global dataset on toxic and potentially toxic metal concentrations in raw goat milk; (ii) elucidate the environmental and agroecological factors driving metal occurrence across rural, urban, and industrial contexts; and (iii) evaluate consumer exposure against international dietary reference thresholds established by the World Health Organization (WHO), the European Food Safety Authority (EFSA), and the Institute of Medicine (IOM).

## 2 Materials and Methods

### 2.1 Data Sources and Search Strategy

We adhered to the PRISMA (Preferred Reporting Items for Systematic Reviews and Meta‐Analyses) guidelines in this systematic review to ensure a rigorous and transparent methodology (Page et al. 2021). A comprehensive literature search was conducted across four major databases: PubMed, Web of Science, Scopus, and Embase. The authors A.C.O.A. and P.A.R. were responsible for selecting the articles and reviewing the selection process to maintain the integrity of the study. The search strategy was meticulously designed to capture relevant studies using the following components: specific keywords, inclusion and exclusion criteria, and Boolean operators to refine the search results. Each stage of the literature search was documented to ensure reproducibility and transparency in line with systematic review standards. The search strategy was meticulously designed to capture relevant studies using the following components:

- Search Component 1 (SC1)—Population search: (goat OR caprine) AND (raw OR fresh) AND (milk);
- Search Component 2 (SC2)—Intervention Search: (heavy metal OR trace elements OR toxic elements OR potentially toxic elements OR mercury OR arsenic OR lead OR cadmium OR chromium OR nickel OR copper OR zinc OR iron OR aluminum OR manganese);
- Search Component 3 (SC3)—Control: concentration OR levels OR absorption;
- Search Component 4 (SC4)—Observation: risk AND assessment OR hazard AND quotient.

The Boolean operator “AND” combined the SC1, SC2, SC3, and SC4 results. This approach ensured a focused and thorough collection of relevant studies for the systematic review.

The research, conducted between May and July 2024, focused exclusively on articles published in English. The inclusion criteria were limited to studies that quantified and detected toxic and potentially toxic metals in raw goat milk. Studies were excluded if they involved processed goat milk or employed experimental modifications to the environment, such as supplementation with elements, to observe changes in the metal composition of raw goat milk. Additionally, research analyzing milk from ruminants other than goats and editorials, letters, reviews, minireviews, and Ph.D. these were not considered.

### 2.2 Focus Question

What are the quantified concentrations of toxic and potentially toxic metals in raw goat milk, and how do these levels correlate with environmental factors and potential health risks in different geographical regions?

Metal concentrations and environmental correlations:

- What are the quantified concentrations of toxic and potentially toxic metals in raw goat milk?
- How do these levels correlate with environmental factors, such as industrial activity, agricultural practices, and water quality?

Geographical variability:

- Which geographic regions show higher concentrations of toxic metals in raw goat milk, and what environmental or anthropogenic factors contribute to this variability?

Health risks:

- What are these metals’ estimated daily intake (EDI) for different populations?
- What are the potential health risks, including non‐carcinogenic effects, for consumers exposed to these metals through raw goat milk?
- What are the carcinogenic risks associated with prolonged exposure to these metals?

Prevalent metals:

- Which toxic and potentially toxic metals are most frequently found in raw goat milk, and what are their primary sources?

### 2.3 Risk of Bias Assessment

A rigorous risk of bias assessment was conducted to ensure this review's methodological robustness and validity. Potential sources of bias were addressed throughout all stages of study selection and synthesis, including the search strategy design, database selection, and eligibility criteria.

An initial scoping search was performed across multiple databases and languages to minimize selection bias, covering diverse article types, years, and indexing platforms. This helped identify thematic gaps and refine targeted inclusion and exclusion criteria aligned with the review's objectives. Language and publication bias were also considered. Although the initial search applied no restrictions, only English‐language articles were retained at full‐text screening due to resource constraints, a limitation acknowledged in Table 1.

**TABLE 1:** Checklist of potential methodological biases.

Domain	Bias category	Description	Mitigation strategy
Search strategy	Selection bias	Use of limited databases, filters, or keywords may exclude relevant studies	Conducted an exploratory scoping search across multiple databases with broad descriptors
Search strategy	Language bias	Restrictions on English‐language publications may exclude important regional studies	The initial search included all languages; the final inclusion was restricted to English with caveats
Search strategy	Publication bias	The exclusion of gray literature may skew the results of studies with positive findings	Only peer‐reviewed studies were included for quality assurance; limitations acknowledged
Eligibility criteria	Inclusion/Exclusion bias	Overly narrow or unclear criteria may omit relevant data or introduce inconsistency	Criteria refined after exploratory phase; transparently aligned with study objectives
Data extraction	Reporting bias	Inconsistent or incomplete reporting of metal concentrations and analytical methods across studies	Only studies with clearly reported quantitative data and methods were extracted
Risk assessment reconstruction	Misclassification bias	Recalculations of EDI, THQ, HI, or CRA from raw data may introduce errors or deviations from original assumptions	Standardized equations and regulatory values (e.g., EPA, WHO) were applied consistently
Measurement bias	Analytical method variability	Different studies used instruments (ICP‐MS, AAS, etc.), which may affect comparability	Discussed in synthesis; no weighting was applied due to heterogeneity
Unit harmonization	Conversion bias	Errors in standardizing concentration units (e.g., mg/L to mg/L) or body weight assumptions can distort risk estimates	All units are converted to mg/L; child/adult weights are standardized (15/70 kg)
Outlier handling	Skewing from implausible values	Excessively high or low concentration values can distort cumulative metrics and risk estimates	Outliers were critically examined and, when necessary, excluded or interpreted cautiously
Geographic representation	Coverage bias	Underrepresenting key regions (e.g., Latin America, Sub‐Saharan Africa) limits generalizability	Limitation explicitly addressed; calls for future regional research included

**^Abbreviations:^ :**CRA, carcinogenic risk assessment; EDI, estimated daily intake; HI, hazard index; THQ, target hazard quotient; WHO, World Health Organization.

Studies were further evaluated for consistency in reporting analytical methods, unit standardization, and clarity in sampling conditions. Discrepant or biologically implausible values were scrutinized and, if necessary, excluded or contextualized in the discussion to avoid skewing results. A transparent and pre‐defined protocol guided the process to enhance reproducibility, minimize bias, and ensure that the synthesized evidence accurately reflects current knowledge on toxic and essential metal exposure through raw goat milk.

### 2.4 Assessment of Metal Ingestion From Raw Goat Milk

The EDI of toxic metals through the consumption of raw goat milk was calculated using the following formula (Meshref et al. 2014):

$$\mathrm{EDI}=\frac{\left(C\times D\right)}{B}$$

where *C* is the concentration of the toxic metal in raw goat milk (mg/L), *D* is the daily consumption of raw goat milk (L/day), *B* is the average body weight of the consumer (kg).

For this study, the average body weight was assumed to be 15 kg for children and 70 kg for adults. Following global dietary data, a daily consumption of 0.25 L/day (250 mL/day) was selected on the basis of nutritional patterns in populations where goat milk is regularly consumed (FAO 2017).

The EDI was calculated for the following toxic and potentially toxic metals: mercury (Hg), arsenic (As), lead (Pb), cadmium (Cd), chromium (Cr), nickel (Ni), copper (Cu), zinc (Zn), iron (Fe), manganese (Mn), and aluminum (Al). This calculation provides a quantitative estimate of exposure to each metal, allowing for the assessment of potential health risks associated with milk consumption. The approach adheres to international standards for dietary exposure evaluations (EFSA 2015; EPA 2023a).

### 2.5 Assessment of Target Hazard Quotient (THQ) for Toxic Metals

THQ was calculated to assess the potential risk associated with the presence of toxic metals in raw goat milk. The THQ is a metric that compares the estimated exposure levels of contaminants with established reference values to determine if the exposure is within safe limits (EPA 2009):

$$\mathrm{THQ}=\frac{\mathrm{EDI}}{\mathrm{RfD}\;}$$

where EDI is the estimated daily intake (mg/L/day), calculated previously, and RfD is the reference dose (mg/L/day) established by regulatory authorities such as the US EPA and EFSA.

The updated RfD values used in this study, based on the most recent assessments, are as follows:

- Mercury (Hg): 0.0001 mg/L/day (EPA 2001);
- Arsenic (As): 0.0003 mg/L/day (EFSA 2012);
- Lead (Pb): No safe threshold; RfD not established (EPAa 2023);
- Cadmium (Cd): 0.0001 mg/L/day (ATSDR 2012);
- Chromium (Cr VI): 0.003 mg/L/day (EPA 1998);
- Nickel (Ni): 0.02 mg/L/day (EPA 1991);
- Copper (Cu): 0.04 mg/L/day (EPA 2003);
- Zinc (Zn): 0.3 mg/L/day (EPA 2005a);
- Iron (Fe): 0.7 mg/L/day (IOM 2001);
- Manganese (Mn): 0.14 mg/L/day (EPA 2011);
- Aluminum (Al): 0.14 mg/L/day (EFSA 2008).

THQ values were interpreted using the following criteria: THQ < 1 indicates that the intake of the toxic metal is within safe levels, whereas THQ ≥ 1 suggests potential health concerns requiring further evaluation (EPA 2009).

### 2.6 Assessment of Hazard Index (HI)

The HI was calculated to evaluate the cumulative non‐carcinogenic risk of multiple toxic metals in raw goat milk. HI is the sum of the individual THQ values and is interpreted as follows:

$$\mathrm{HI}=\sum \mathrm{TH}Q_{i}$$

where THQ_*i*_ represents the target hazard quotient for the *i*th metal.

For this study, the metals considered in the HI calculation included only those metals that are non‐carcinogenic, specifically copper (Cu), zinc (Zn), iron (Fe), manganese (Mn), and aluminum (Al). The HI was calculated for each reference group (children and adults) to assess the potential cumulative risk for each group on the basis of these metals. If the HI value is less than 1 (HI < 1), the combined exposure to toxic metals is considered within safe limits, indicating a low risk of non‐carcinogenic health effects. However, if the HI value is greater than or equal to 1 (HI ≥ 1), this suggests that the combined exposure to toxic metals may pose a significant health risk, requiring further investigation (EPA 2009).

### 2.7 Carcinogenic Risk Assessment (CRA)

The CRA estimates the lifetime probability of developing cancer due to chronic exposure to carcinogenic metals via raw goat milk. CRA was calculated as

CRA=EDI×SF

where EDI is the estimated daily intake (mg/L/day), and SF is the oral slope factor (mg/L/day)^−1^, reflecting the carcinogenic potency of the metal.

Slope factors used in this study were

- Arsenic (As): 1.5 (mg/L/day)^−1^ (EPA 2001);
- Chromium (Cr VI): 0.5 (mg/L/day)^−1^ (EPA 2011).

Cadmium (Cd) and lead (Pb) were excluded from quantitative CRA due to the absence of officially established oral slope factors by regulatory agencies such as the US EPA and ATSDR. CRA values within the range of 10^−6^ to 10^−4^ are considered acceptable or negligible, whereas values above 10^−4^ indicate increased carcinogenic risk and may warrant further attention (EPA 2005b).

## 3 Results

### 3.1 Screening and Selection of Relevant Articles

The extensive search of the four major databases mentioned yielded 841 articles: 90 from Embase, 156 from PubMed, one from Scopus, and 594 from Web of Science. After removing ten duplicates, 831 articles were identified for further evaluation. The first passing was done by checking and carefully reading titles and abstracts so that their relevance to the study on the occurrence of toxic and potentially toxic metals in raw goat milk could be evaluated. This secondarily yielded 33 articles for full‐text review.

**FIGURE 1:**
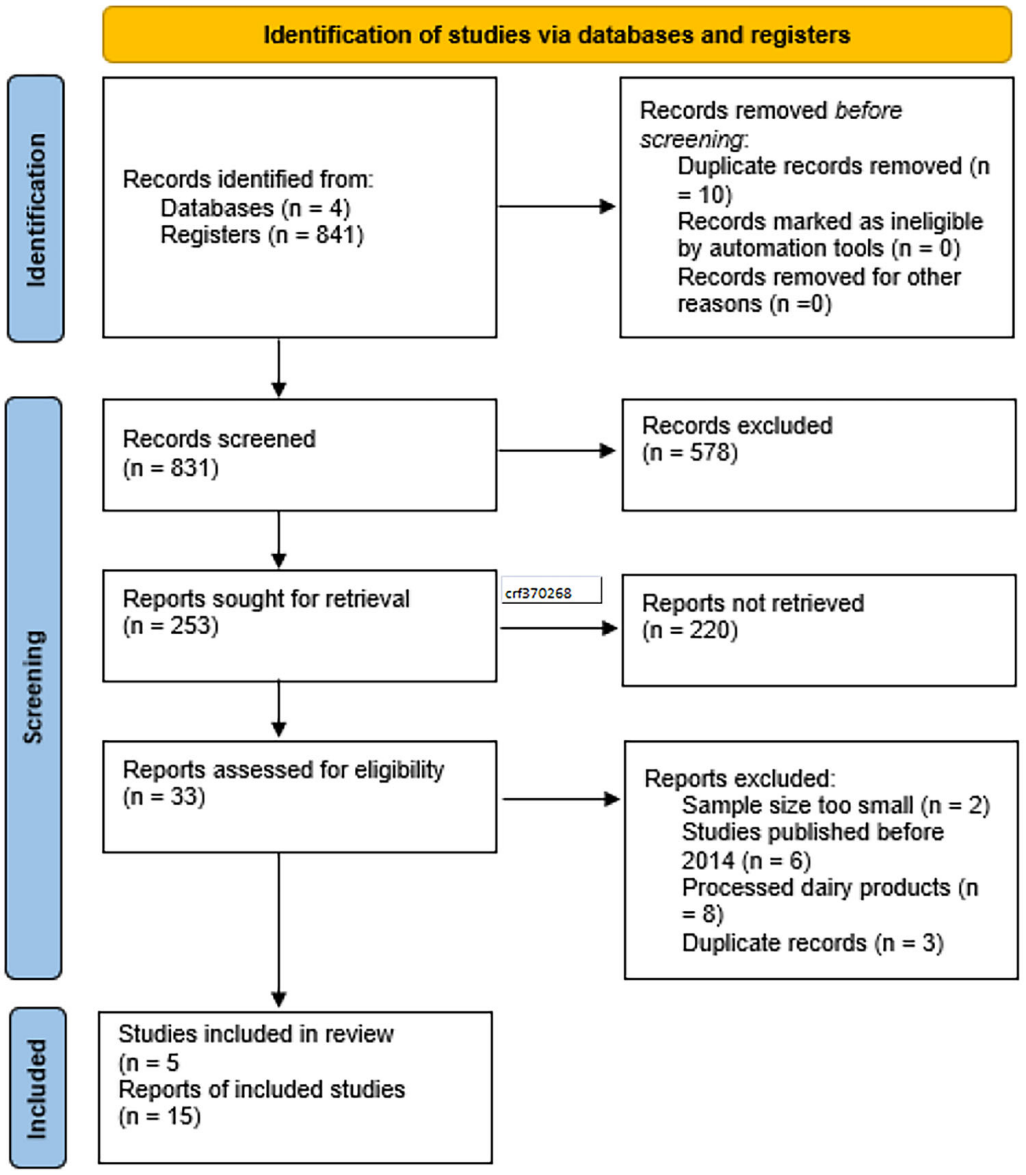
Flow diagram displaying the results of the literature search (PubMed, Embase, Web of Science, and Scopus).

During the subsequent full‐text review, exclusion criteria were applied to discard everything not directed at raw goat milk or presented experimental studies aimed at modifying the concentration of toxic or potentially toxic elements or that did not discriminate the species of small ruminants in the study. This finally streamlined the invocation of 15 articles that met discrete selection criteria (Figure 1).

In conjunction with the aforementioned database search, a manual search was performed to find any other relevant studies that may have been missed during the initial screening. This eventually provided five additional articles to be included in the systematic review. So, by final inclusion, the systematic review had 20 articles (Figure 1).

### 3.2 Toxicologically Relevant Metals in Raw Goat Milk

In this systematic review, we present an integrated assessment of the elemental profile of raw goat milk, recognizing its nutritional importance alongside the potential toxicological hazards of metal contaminants. Table 2 succinctly and rigorously summarizes the mean concentrations ± standard deviations (where available) and observed ranges (mg L^−1^) for four toxic metals, arsenic (As), lead (Pb), cadmium (Cd), and mercury (Hg), as well as six essential trace elements, chromium (Cr), nickel (Ni), copper (Cu), zinc (Zn), iron (Fe), and manganese (Mn).

**TABLE 2:** Concentrations of toxic and potentially toxic elements in raw goat milk across different regions.

Reference	Location	Zone	Concentrations (mean ± SD/mg/L)
Ahmad et al. (2017)	Shnwa Gudi Khel, District Karak, Pakistan	Rural zone	Pb: <LOD; Cd: 0.074 ± 0.003; Cr: 1.152 ± 0.045; Ni: 1.152 ± 0.045; Cu: 0.212 ± 0.010; Zn: 3.345 ± 0.071; Fe: 0.950 ± 0.305; Mn: 0.065 ± 0.032
Almášiová et al. (2023)	Orava, Slovakia	Rural zone	As: <LOD; Pb: <LOD; Cd: <LOD; Al: <LOD; Cr: <LOD; Ni: <LOD; Cu: 0.12 ± 0.02; Zn: <LOD; Fe: 1.68 ± 1.40; Mn: 0.06 ± 0.05
	Stredné Považie, Slovakia	Rural zone	As: <LOD; Pb: <LOD; Cd: <LOD; Al: <LOD; Cr: <LOD; Ni: <LOD; Cu: <LOD; Zn: 2.35 ± 1.46; Fe: 1.74 ± 0.33; Mn: <LOD
Chen et al. (2020)	Shandong and Shaanxi provinces, China	Rural zone	As: 0.04427 ± 0.00393 mg; Pb: 0.00797 ± 0.00750; Cd: 0.000425 ± 0.000305; Al: 0.277 ± 0.155; Cr: 0.0117 ± 0.0052; Ni: 0.0383 ± 0.0263; Cu: 0.208 ± 0.098; Zn: 3.11 ± 0.81; Fe: 1.08 ± 0.38; Mn: 0.156 ± 0.031
El‐Badry and Raslan (2016)	Zagazig city, Sharkia governorate, Egypt	Rural zone	Pb: 0.0122 ± 0.00013; Cu: 0.00086 ± 0.00005
Gougoulias et al. (2014)	Thessaly, Greece	Industrial zone	Pb: <LOD; Cd: 0.12 ± 0.02; Cr: 0.12 ± 0.02; Ni: <LOD
Guo et al. (2021)	Shanxi, China	Rural zone	As: 0.00675 ± 0.00241; Cr: 0.03259 ± 0.04384; Cu: 0.60492 ± 0.30213; Mn: 0.49546 ± 0.46136
	Shandong, China	Rural zone	As: 0.00761 ± 0.00251; Cr: 0.15663 ± 0.16104; Cu: 0.86476 ± 0.49038; Mn: 0.75189 ± 0.31618
	Yunnan, China	Rural zone	As: 0.01094 ± 0.00356; Cr: 0.04484 ± 0.05311; Cu: 0.66275 ± 0.24095; Mn: 0.57258 ± 0.35908
Homayonibezi et al. (2021)	Asaluyeh, Iran	Industrial zone	Pb: 0.141 ± 0.030; Cr: 16.423 ± 0.349; Ni: 1.447 ± 0.101; Cu: 0.146 ± 0.118; Fe: 6.111 ± 0.501; Mn: 0.239 ± 0.016
	Kaki, Iran	Rural zone	Pb: <LOD; Cr: 14.211 ± 0.205; Ni: 1.319 ± 0.036; Cu: 0.171 ± 0.143; Fe: 9.135 ± 0.558; Mn: 0.257 ± 0.027
Ismail et al. (2015)	North West Multan, Pakistan	Residencial zone	Pb: 0.009 ± 0.001; Cd: <LOD; Ni: 0.014 ± 0.002; Cu: 0.244 ± 0.03
	South East Multan, Pakistan	Residencial zone	Pb: 0.013 ± 0.002; Cd: <LOD; Ni: 0.014 ± 0.001; Cu: 0.453 ± 0.04
	North East Multan, Pakistan	Residencial zone	Pb: <LOD; Cd: 0.0043 ± 0.002; Ni: <LOD; Cu: 0.340 ± 0.02
	Southwest Multan, Pakistan	Industrial zone	Pb: 0.009 ± 0.001; Cd: <LOD; Ni: 0.012 ± 0.003; Cu: 0.693 ± 0.05
Kandhro et al. (2023)	Tharparkar, Pakistan	Rural zone	As: 0.0325 ± 0.0012; Pb: 0.0367 ± 0.0035; Cd: 0.0163 ± 0.00054
	Hyderabad city, Pakistan	Rural zone	As: 0.124 ± 0.00082; Pb: 0.0132 ± 0.00083; Cd: 0.00356 ± 0.00011
Korac et al. (2023)	Herzegovina, Bosnia and Herzegovina	Rural zone	Cu: 0.21 ± 0.13; Zn: 2.80 ± 1.15
	Sarajevo, Bósnia and Herzegovina, Bosnia and Herzegovina	Rural zone	Cu: 0.90 ± 0.52; Zn: 4.40 ± 0.76
Lauková et al. (2022)	Eastern Slovakia, Slovakia	Rural zone	Cu: 0.1746 ± 0.0.0463; Zn: 2.561 ± 0.6823; Fe: 1.383 ± 0.5087; Mn: 0.051 ± 0.0238
Lebsir et al. (2023)	El Milia, Algeria	Industrial zone	Pb: 0.0425 ± 0.0065; Cd: 0.0155 ± 0.0021; Cu: 0.0500 ± 0.0024; Zn: 1.425 ± 0.1042
	Texenna, Algeria	Rural zone	Pb: 0.382 ± 0.0026; Cd: 0.00397 ± 0.0007; Cu: 0.0633 ± 0.0044; Zn: 2.712 ± 0.1148
	Djimla, Algeria	Rural zone	Pb: 0.338 ± 0.0223; Cd: 0.0359 ± 0.0025; Cu: 0.0369 ± 0.0019; Zn: 1.467 ± 0.1298
Miedico et al. (2016)	Manfredonia and Brindisi, Italy	Industrial zone	As: 0.00510 ± 0.0010; Pb: 0.00460 ± 0.00320; Cd: 0.000650 ± 0.000390; Al: 0.250 ± 0.210; Cr: 0.00210 ± 0.00180; Ni: 0.0440 ± 0.0350; Cu: 0.0770 ± 0.0490; Zn: 3.500 ± 1.400; Fe: 0.850 ± 0.240; Mn: 0.0690 ± 0.0310
Osório et al. (2015)	Anogyra, Chipre, Cyprus	Rural zone	Al: <LOD; Zn: 1.77 ± 0.45; Fe: 0.14 ± 0.05; Mn: 0.09 ± 0.04
	Cyprus, Chipre, Cyprus	Rural zone	Al: <LOD; Zn: 1.85 ± 0.71; Fe: 0.32 ± 0.21; Mn: 0.05 ± 0.02
Osório et al. (2015)	Kofinou, Chipre, Cyprus	Rural zone	Al: 1.24 ± 0.35; Zn: 1.24 ± 0.22; Fe: 0.16 ± 0.07; Mn: 0.04 ± 0.01
Parsaei et al. (2019)	Isfahan Province, Iran, Iran	Rural zone	Hg: 0.00510 ± 0.00050; Pb: 0.01025 ± 0.00101; Cd: 0.00322 ± 0.00031
Singh et al. (2014)	Hisar, India	Rural zone	Cu: <LOD; Zn: 5.1 ± 1.7; Fe: 9.1 ± 5.5
Singh et al. (2019)	Karnal, India	Rural zone	Cu: 0.50 ± 0.1; Zn: 4.8 ± 6.7; Fe: 0.78 ± 0.14; Mn: 0.3 ± 0.12
Wanniatie et al. (2019)	Bagor, Indonesia	Rural zone	As: 0.07 ± 0.00013; Pb: 0.050 ± 0.00013
	Bagor, Indonesia	Rural zone	As: 0.11 ± 0.00019; Pb: 0.08 ± 0.00013
Zhou et al. (2017)	Shaanxi and Shandong, China	Rural zone	Hg: 0.00011–0.00011; As: 0.00005–0.00044; Pb: 0.00152–0.00240; Cd: 0.00–0.00002; Al: 0.06940–0.13862; Cr: 0.00041–0.00236; Ni: 0.00175–0.00584; Cu: 0.08152–0.11330; Zn: 2.98343–3.28177; Fe: 0.36115–0.52519; Mn: 0.03378–0.04026

*Note*: Concentrations are presented as reported initially or converted to a standard unit (mg/L) for comparability. According to the authors’ reporting, values are expressed as means ± standard deviation, ranges, or detection limits. All values reported as below the detection limit (e.g., <0.02, <0.0001, or BDL) were standardized and are denoted as <LOD. Elements not analyzed by a given study were excluded from the summary. The table includes the following elements when available: mercury (Hg), arsenic (As), lead (Pb), cadmium (Cd), aluminum (Al), chromium (Cr), nickel (Ni), copper (Cu), zinc (Zn), iron (Fe), and manganese (Mn). Zhou et al. (2017) reported values corresponding to the median and 75th percentile (P75), not mean ± SD.

#### 3.2.1 Toxic Metals

##### 3.2.1.1 Mercury (Hg)

This review identified only two studies investigating mercury concentrations in raw goat's milk. Among the available data (Table 2), the concentrations ranged from 0.00510 ± 0.00050 to 0.00011 mg/L. Geographically, the highest concentration was observed in Isfahan Province, Iran (Parsaei et al. 2019), whereas the lowest was reported in Shaanxi and Shandong Provinces in China (Zhou et al. 2017).

##### 3.2.1.2 Arsenic (As)

Arsenic concentrations reported in raw goat milk ranged from <LOD in Slovakia (Almášiová et al. 2023) to 0.124 ± 0.00082 mg/L in Hyderabad, Pakistan (Kandhro et al. 2023). Notable concentrations were also observed in conventional farms in Indonesia (Wanniatie et al. 2019), with values reaching up to 0.11 ± 0.00019 mg/L (Table 2). In Europe, Almášiová et al. (2023) found no detectable arsenic in Slovakian conventional farms, whereas Miedico et al. (2016) detected 0.00510 ± 0.0010 mg/L in industrial areas of Italy near landfills and power plants. In Asia, Zhou et al. (2017) reported 0.00005–0.00044 mg/L in Shaanxi and Shandong, and Chen et al. (2020) documented 0.04427 ± 0.00393 mg/L in rural areas of the same provinces. Guo et al. (2021) measured intermediate concentrations of 0.00675 ± 0.00241 mg/L in Shanxi, 0.00761 ± 0.00251 mg/L in Shandong, and 0.01094 ± 0.00356 mg/L in Yunnan. In Southeast Asia, Wanniatie et al. (2019) recorded concentrations in conventional farms, with values up to 0.11 ± 0.00019 mg/L, comparable to the upper limit observed in Pakistan.

##### 3.2.1.3 Lead (Pb)

Lead (Pb) concentrations reported in raw goat milk ranged from <LOD in Slovakia (Almášiová et al. 2023) to 0.382 ± 0.0026 mg/L in Texenna, Algeria (Lebsir et al. 2023) (Table 2). In Europe, Almášiová et al. (2023) found no detectable Pb in Slovak farms, both in rural Orava and Stredné Považie. Additionally, Gougoulias et al. (2014) also measured <LOD in an industrial zone in Thessaly, Greece.

In Asia, Ahmad et al. (2017) documented levels below detection in Karak District, Pakistan. Ismail et al. (2015) reported <LOD in northeastern residential sectors of Multan, Pakistan, but detected 0.013 ± 0.002 mg/L in southeastern residential zones and 0.009 ± 0.001 mg/L in a southwestern industrial sector. Kandhro et al. (2023) recorded intermediate concentrations of 0.0132 ± 0.00083 mg/L in Hyderabad and 0.0367 ± 0.0035 mg/L in Tharparkar, Pakistan. Further west in Asia, El‑Badry and Raslan (2016) found 0.0122 ± 0.00013 mg/L in Zagazig, Egypt, whereas Parsaei et al. (2019) reported 0.01025 ± 0.00101 mg/L in Isfahan Province, Iran. Homayonibezi et al. (2021) also reported Pb levels in Iran, with 0.141 ± 0.030 mg/L in industrial Asaluyeh and <LOD in rural Kaki.

In Southeast Asia, Wanniatie et al. (2019) detected Pb in Indonesia, measuring 0.050 ± 0.00013 and 0.08 ± 0.00013 mg/L in rural areas of Bagor. In North Africa, Lebsir et al. (2023) documented the highest concentrations in Algeria. The single highest Pb concentration observed across all regions and studies was 0.382 ± 0.0026 mg/L in Texenna (rural zone), surpassing 0.338 ± 0.0223 mg/L in Djimla (rural) and 0.0425 ± 0.0065 mg/L in El Milia (industrial) (Table 2).

##### 3.2.1.4 Cadmium (Cd)

Cadmium (Cd) concentrations in raw goat milk, as reported by multiple studies, covered a broad spectrum, ranging from <LOD on rural farms in Orava and Stredné Považie, Slovakia (Almášiová et al. 2023) to a peak value of 0.12 ± 0.02 mg/L recorded in industrial zones of Thessaly, Greece, by Gougoulias et al. (2014) (Table 2). In Europe, beyond the <LOD findings by Almášiová et al. (2023), Miedico et al. (2016) measured 0.000650 ± 0.000390 mg/L in the industrial regions of Manfredonia and Brindisi, Italy. However, the highest European concentration was that reported by Gougoulias et al. (2014) in the Greek industrial zone.

In Asia, Ahmad et al. (2017) documented levels of 0.074 ± 0.003 mg/L in Shnwa Gudi Khel, a rural district of Pakistan. Ismail et al. (2015) reported <LOD for Cd in several residential (northwestern and southeastern) and industrial (southwestern) sectors of Multan, Pakistan, though they detected 0.0043 ± 0.002 mg/L in the northeastern residential sector. Kandhro et al. (2023) later recorded intermediate levels of 0.0163 ± 0.00054 mg/L in rural Tharparkar and 0.00356 ± 0.00011 mg/L in rural Hyderabad, Pakistan. In Iran, Parsaei et al. (2019) observed 0.00322 ± 0.00031 mg/L in the rural province of Isfahan.

In East Asia (China), Zhou et al. (2017) documented Cd levels ranging from 0.00 to 0.00002 mg/L in rural Shaanxi and Shandong Provinces, whereas Chen et al. (2020) identified 0.000425 ± 0.000305 mg/L in rural Shandong and Shaanxi. In North Africa, specifically Algeria, Lebsir et al. (2023) reported a range of Cd concentrations: 0.00397 ± 0.0007 mg/L in Texenna (rural zone), 0.0155 ± 0.0021 mg/L in El Milia (industrial zone), and the region's highest value of 0.0359 ± 0.0025 mg/L in Djimla (rural zone).

##### 3.2.1.5 Aluminum (Al)

Aluminum (Al) concentrations in raw goat milk, as documented by five studies, displayed a wide span of values, ranging from <LOD on rural farms in Orava and Stredné Považie, Slovakia (Almášiová et al. 2023), to a maximum of 1.24 ± 0.35 mg/L in Kofinou, Cyprus, as reported by Osório et al. (2015) (Table 2).

In Europe, beyond the <LOD findings by Almášiová et al. (2023) in Slovak rural systems, Miedico et al. (2016) measured 0.250 ± 0.210 mg/L in industrial zones of Manfredonia and Brindisi, Italy. Osório et al. (2015) recorded the highest regional levels, with 1.24 ± 0.35 mg/L in Kofinou, whereas other Cypriot locations, such as Anogyra and Cyprus, showed <LOD. In Asia, Zhou et al. (2017) reported concentrations ranging from 0.06940 to 0.13862 mg/L in rural Shaanxi and Shandong Provinces, China. Under comparable rural conditions, Chen et al. (2020) found mean levels of 0.277 ± 0.155 mg/L in Shandong and Shaanxi.

#### 3.2.2 Essential Trace Metals (Potentially Harmful at High Concentrations)

##### 3.2.2.1 Chromium (Cr)

As documented by eight studies, chromium (Cr) levels in raw goat milk exhibited pronounced variability, ranging from 0.00041 to 16.423 mg/L across the reviewed literature (Table 2). In Europe, Miedico et al. (2016) measured just 0.00210 ± 0.00180 mg/L in Manfredonia and Brindisi, Italy, despite the proximity to waste facilities and power plants. Gougoulias et al. (2014) reported 0.12 ± 0.02 mg/L in Thessaly, Greece, whereas Almášiová et al. (2023) found <LOD concentrations on rural farms in Orava and Stredné Považie, Slovakia.

In East Asia (China), Zhou et al. (2017) reported a range of 0.00041–0.00236 mg/L in rural Shaanxi and Shandong, whereas Chen et al. (2020) identified 0.0117 ± 0.0052 mg/L under mixed agricultural management in the same provinces. Guo et al. (2021) observed substantially higher and more variable levels, reaching 0.04484 ± 0.05311 mg/L in Yunnan, 0.15663 ± 0.16104 mg/L in Shandong, and 0.03259 ± 0.04384 mg/L in Shanxi. In South Asia, Ahmad et al. (2017) documented 1.152 ± 0.045 mg/L in Shnwa Gudi Khel, Karak District, Pakistan, attributing contamination to nearby mining and metallurgical industries. In the Middle East, Homayonibezi et al. (2021) recorded extreme contamination in the petrochemical zone of Asaluyeh, Iran, with Cr concentrations of 16.423 ± 0.349 mg/L at one location and 14.211 ± 0.205 mg/L at a nearby “control” rural site in Kaki.

##### 3.2.2.2 Nickel (Ni)

As documented by nine distinct studies, nickel (Ni) concentrations in raw goat milk showed a broad range, spanning from 0.00175 to 1.447 mg/L across the reviewed datasets. In Europe, Gougoulias et al. (2014) reported <LOD values for Ni in Thessaly, Greece. In contrast, Miedico et al. (2016) observed 0.0440 ± 0.0350 mg/L in industrial zones of Manfredonia and Brindisi, Italy. On rural Slovak farms, specifically in Orava and Stredné Považie, Almášiová et al. (2023) also recorded Ni concentrations <LOD.

In East Asia (China), Zhou et al. (2017) measured values ranging from 0.00175 to 0.00584 mg/L in rural areas of Shaanxi and Shandong Provinces, whereas Chen et al. (2020) documented 0.0383 ± 0.0263 mg/L in rural Shandong and Shaanxi. In South Asia (Pakistan), Ismail et al. (2015) reported Ni levels ranging from <LOD in the northeastern residential sector of Multan to 0.014 ± 0.002 mg/L in the northwestern residential sector, 0.014 ± 0.001 mg/L in the southeastern residential sector, and 0.012 ± 0.003 mg/L in the southwestern industrial industry. Additionally, Ahmad et al. (2017) found 1.152 ± 0.045 mg/L in Shnwa Gudi Khel, Karak District, a rural area where contamination was linked to nearby industrial activities. In the Middle East (Iran), Homayonibezi et al. (2021) reported the highest concentrations, with 1.447 ± 0.101 mg/L in the petrochemical industrial zone of Asaluyeh and 1.319 ± 0.036 mg/L in Kaki, a nearby rural area.

##### 3.2.2.3 Copper (Cu)

Copper (Cu) concentrations, as reported across fourteen studies, displayed substantial variability, spanning from <LOD in Hisar, India (Singh et al. 2014) to a peak of 0.90 ± 0.52 mg/L in Sarajevo, Bosnia and Herzegovina (Korac et al. 2023) (Table 2). In Europe, Almášiová et al. (2023) found Cu levels of 0.12 ± 0.02 mg/L in Orava and <LOD in Stredné Považie on rural Slovak farms. Korac et al. (2023) documented 0.90 ± 0.52 mg/L in Sarajevo and 0.21 ± 0.13 mg/L in Herzegovina, revealing substantial regional variation. Lauková et al. (2022) observed 0.1746 ± 0.0463 mg/L in Eastern Slovakia, whereas Miedico et al. (2016) measured 0.0770 ± 0.0490 mg/L in industrial zones of Manfredonia and Brindisi, Italy.

In East Asia (China), Zhou et al. (2017) reported a range of 0.08152–0.11330 mg/L in rural areas of Shaanxi and Shandong. Guo et al. (2021) found markedly higher averages, with 0.60492 ± 0.30213 mg/L in Shanxi, 0.66275 ± 0.24095 mg/L in Yunnan, and 0.86476 ± 0.49038 mg/L in Shandong. Chen et al. (2020) recorded a mean of 0.208 ± 0.098 mg/L in rural Shandong and Shaanxi. In the Middle East (Iran), Homayonibezi et al. (2021) measured 0.146 ± 0.118 mg/L in the industrial zone of Asaluyeh and 0.171 ± 0.143 mg/L in the rural area of Kaki.

Across South Asia (Pakistan and India), Ismail et al. (2015) reported concentrations of 0.244 ± 0.03 mg/L in the northwestern residential sector of Multan, 0.453 ± 0.04 mg/L in the southeastern residential sector, 0.340 ± 0.02 mg/L in the northeastern residential sector, and 0.693 ± 0.05 mg/L in the southwestern industrial sector. Ahmad et al. (2017) documented 0.212 ± 0.010 mg/L in Shnwa Gudi Khel, Karak District, Pakistan. Singh et al. (2014) found <LOD in Hisar, India, whereas Singh et al. (2019) reported 0.50 ± 0.1 mg/L in Karnal, India. In North Africa (Algeria), Lebsir et al. (2023) recorded concentrations of 0.0500 ± 0.0024 mg/L in El Milia (industrial zone), 0.0633 ± 0.0044 mg/L in Texenna (rural zone), and 0.0369 ± 0.0019 mg/L in Djimla (rural zone). Finally, in Egypt, El‑Badry and Raslan (2016) reported 0.00086 ± 0.00005 mg/L in Zagazig City.

##### 3.2.2.4 Zinc (Zn)

Zinc concentrations reported across nine studies ranged from < LOD in ecological Slovak farms (Almášiová et al. 2023) to 5.1 ± 1.7 mg/L in Hisar, India (Singh et al. 2015) (Table 3). In Europe, Almášiová et al. (2023) found no detectable Zn in ecological farms, whereas conventional systems averaged 2.35 ± 1.46 mg/L. Lauková et al. (2022) documented 2.561 ± 0.6823 mg/L in Eastern Slovakia, and Korac et al. (2023) observed 2.80 ± 1.15 mg/L in Herzegovina, with markedly higher concentrations in Sarajevo (4.40 ± 0.76 mg/L). Osório et al. (2015) measured 1.24 ± 0.22 to 1.85 ± 0.71 mg/L in Anogyra and Kofinou, Cyprus. In East Asia, Zhou et al. (2017) reported 2.98343–3.28177 mg/L in Shaanxi and Shandong, whereas Chen et al. (2020) found a comparable average of 3.11 ± 0.81 mg/L. In South Asia, Singh et al. (2019) documented 4.8 ± 6.7 mg/L in Karnal, India. In Southern Europe, industrial zones in Italy showed an average of 3.500 ± 1.400 mg/L (Miedico et al. 2016).

**TABLE 3:** Estimated daily intake (EDI) of toxic and potentially toxic elements through consumption of raw goat milk.

Reference	Location	Zone	EDI (Adult/Child)
Ahmad et al. (2017)	Shnwa Gudi Khel, District Karak, Pakistan	Rural zone	Cd: 2.64e − 04/1.23e − 03; Cr: 4.11e − 03/1.92e − 02; Ni: 4.11e − 03/1.92e − 02; Cu: 7.57e − 04/3.53e − 03; Zn: 1.19e − 02/5.58e − 02; Fe: 3.39e − 03/1.58e − 02; Mn: 2.32e − 04/1.08e − 03
Almášiová et al. (2023)	Orava, Slovakia	Rural zone	Cu: 4.29e − 04/2.00e − 03; Fe: 6.00e − 03/2.80e − 02; Mn: 2.14e − 04/1.00e − 03
	Stredné Považie, Slovakia	Rural zone	Zn: 8.39e − 03/3.92e − 02; Fe: 6.21e − 03/2.90e − 02
Chen et al. (2020)	Shandong and Shaanxi province, China	Rural zone	As: 1.58e − 04/7.38e − 04; Pb: 2.85e − 05/1.33e − 04; Cd: 1.52e − 06/7.08e − 06; Cr: 4.18e − 05/1.95e − 04; Ni: 1.37e − 04/6.38e − 04; Cu: 7.43e − 04/3.47e − 03; Zn: 1.11e − 02/5.18e − 02; Fe: 3.86e − 03/1.80e − 02; Mn: 5.57e − 04/2.60e − 03
El‐Badry and Raslan (2016)	Zagazig city, Sharkia governorate, Egypt	Rural zone	Pb: 4.36e − 05/2.03e − 04; Cu: 3.07e − 06/1.43e − 05
Gougoulias et al. (2014)	Greece, Thessaly	Industrial zone	Cd: 4.29e − 04/2.00e − 03; Cr: 4.29e − 04/2.00e − 03
Guo et al. (2021)	Manfredonia and Brindisi, Italy	Industrial zone	As: 1.82e − 05/8.50e − 05; Pb: 1.64e − 05/7.67e − 05; Cd: 2.32e − 06/1.08e − 05; Al: 8.93e − 04/4.17e − 03; Cr: 7.50e − 06/3.50e − 05; Ni: 1.57e − 04/7.33e − 04; Cu: 2.75e − 04/1.28e − 03; Zn: 1.25e − 02/5.83e − 02; Fe: 3.04e − 03/1.42e − 02; Mn: 2.46e − 04/1.15e − 03
	Shandong, China	Rural zone	As: 2.72e − 05/1.27e − 04; Cr: 5.59e − 04/2.61e − 03; Cu: 3.09e − 03/1.44e − 02; Mn: 2.69e − 03/1.25e − 02
	Shanxi, China	Rural zone	As: 2.41e − 05/1.12e − 04; Cr: 1.16e − 04/5.43e − 04; Cu: 2.16e − 03/1.01e − 02; Mn: 1.77e − 03/8.26e − 03
Homayonibezi et al. (2021)	Yunnan, China	Rural zone	As: 3.91e − 05/1.82e − 04; Cr: 1.60e − 04/7.47e − 04; Cu: 2.37e − 03/1.10e − 02; Mn: 2.04e − 03/9.54e − 03
	Asaluyeh, Iran	Industrial zone	Pb: 5.04e − 04/2.35e − 03; Cr: 5.87e − 02/2.74e − 01; Ni: 5.17e − 03/2.41e − 02; Cu: 5.21e − 04/2.43e − 03; Fe: 2.18e − 02/1.02e − 01; Mn: 8.54e − 04/3.98e − 03
Ismail et al. (2015)	Kaki, Iran	Rural zone	Cr: 5.08e − 02/2.37e − 01; Ni: 4.71e − 03/2.20e − 02; Cu: 6.11e − 04/2.85e − 03; Fe: 3.26e − 02/1.52e − 01; Mn: 9.18e − 04/4.28e − 03
	North West Multan, Pakistan	Residencial zone	Pb: 3.21e − 05/1.50e − 04; Ni: 5.00e − 05/2.33e − 04; Cu: 8.71e − 04/4.07e − 03
	North east Multan, Pakistan	Residencial zone	Cd: 1.54e − 05/7.17e − 05; Cu: 1.21e − 03/5.67e − 03
	South east Multan, Pakistan	Residencial zone	Pb: 4.64e − 05/2.17e − 04; Ni: 5.00e − 05/2.33e − 04; Cu: 1.62e − 03/7.55e − 03
Kandhro et al. (2023)	South west Multan, Pakistan	Industrial zone	Pb: 3.21e − 05/1.50e − 04; Ni: 4.29e − 05/2.00e − 04; Cu: 2.47e − 03/1.15e − 02
	Hyderabad, Pakistan	Rural zone	As: 4.43e − 04/2.07e − 03; Pb: 4.71e − 05/2.20e − 04; Cd: 1.27e − 05/5.93e − 05
Korac et al. (2023)	Tharparkar, Pakistan	Rural zone	As: 1.16e − 04/5.42e − 04; Pb: 1.31e − 04/6.12e − 04; Cd: 5.82e − 05/2.72e − 04
	Herzegovina, Pakistan	Rural zone	Cu: 7.50e − 04/3.50e − 03; Zn: 1.00e − 02/4.67e − 02
Lauková et al. (2022)	Herzegovina, Bosnia and Herzegovina	Rural zone	Cu: 3.21e − 03/1.50e − 02; Zn: 1.57e − 02/7.33e − 02
Lebsir et al. (2023)	Eastern Slovakia	Rural zone	Cu: 6.24e − 04/2.91e − 03; Zn: 9.15e − 03/4.27e − 02; Fe: 4.94e − 03/2.31e − 02; Mn: 1.82e − 04/8.50e − 04
	Djimla, Algeria	Rural zone	Pb: 1.21e − 03/5.63e − 03; Cd: 1.28e − 04/5.98e − 04; Cu: 1.32e − 04/6.15e − 04; Zn: 5.24e − 03/2.45e − 02
	El Milia, Algeria	Industrial zone	Pb: 1.52e − 04/7.08e − 04; Cd: 5.54e − 05/2.58e − 04; Cu: 1.79e − 04/8.33e − 04; Zn: 5.09e − 03/2.38e − 02
Miedico et al. (2016)	Texenna, Algeria	Rural zone	Pb: 1.36e − 03/6.37e − 03; Cd: 1.42e − 05/6.62e − 05; Cu: 2.26e − 04/1.05e − 03; Zn: 9.69e − 03/4.52e − 02
Osório et al. (2015)	Anogyra, Chipre	Rural zone	Zn: 6.32e − 03/2.95e − 02; Fe: 5.00e − 04/2.33e − 03; Mn: 3.21e − 04/1.50e − 03
	Cypirus, Chipre	Rural zone	Zn: 6.61e − 03/3.08e − 02; Fe: 1.14e − 03/5.33e − 03; Mn: 1.79e − 04/8.33e − 04
Osório et al. (2015)	Kofinou, Chipre	Rural zone	Al: 4.43e − 03/2.07e − 02; Zn: 4.43e − 03/2.07e − 02; Fe: 5.71e − 04/2.67e − 03; Mn: 1.43e − 04/6.67e − 04
Parsaei et al. (2019)	Isfahan Province, Iran	Rural zone	Hg: 1.82e − 05/8.50e − 05; Pb: 3.66e − 05/1.71e − 04; Cd: 1.15e − 05/5.37e − 05
Singh et al. (2014)	Hisar, India	Rural zone	Zn: 1.82e − 02/8.50e − 02; Fe: 3.25e − 02/1.52e − 01
Singh et al. (2019)	Karnal, India	Rural zone	Cu: 1.79e − 03/8.33e − 03; Zn: 1.71e − 02/8.00e − 02; Fe: 2.79e − 03/1.30e − 02; Mn: 1.07e − 03/5.00e − 03
Wanniatie et al. (2019)	Bagor, Indonesia	Rural zone	As: 2.50e − 04/1.17e − 03; Pb: 1.79e − 04/8.33e − 04; As: 3.93e − 04/1.83e − 03; Pb: 2.86e − 04/1.33e − 03
Zhou et al. (2017)	Shaanxi and Shandong, China	Rural zone	Hg: 3.93e − 07/1.83e − 06; As: 1.79e − 07/8.33e − 07; Pb: 5.43e − 06/2.53e − 05; Cd: 0.00e + 00/0.00e + 00; Al: 2.48e − 04/1.16e − 03; Cr: 1.46e − 06/6.83e − 06; Ni: 6.25e − 06/2.92e − 05; Cu: 2.91e − 04/1.36e − 03; Zn: 1.07e − 02/4.97e − 02; Fe: 1.29e − 03/6.02e − 03; Mn: 1.21e − 04/5.63e − 04

*Note*: Values represent the estimated daily intake (EDI) of metals for adult and pediatric populations, expressed in scientific notation (Adult/Child, mg/l body weight/day). The EDI was calculated on the basis of measured concentrations in raw goat milk and standard consumption/body weight assumptions.

##### 3.2.2.5 Iron (Fe)

Iron concentrations in raw goat milk spanned from 0.14 ± 0.05 mg/L in Cyprus (Osório et al. 2015) to 9.135 ± 0.558 mg/L in Kaki, Iran (Homayonibezi et al. 2021) (Table 3). In Europe, Osório et al. (2015) reported 0.14–0.16 mg/L in Cyprus, whereas Lauková et al. (2022) measured 1.383 ± 0.5087 mg/L in Eastern Slovakia, with variations linked to grazing and lactation. Miedico et al. (2016) found intermediate levels (0.850 ± 0.240 mg/L) in industrial areas of Italy. In East Asia, Zhou et al. (2017) documented 0.36115–0.52519 mg/L in rural Shaanxi and Shandong, and Chen et al. (2020) reported 1.08 ± 0.38 mg/L under more intensive agricultural systems. In South Asia, Singh et al. (2015) observed 9.1 ± 5.5 mg/L in Hisar, India, whereas Ahmad et al. (2017) found 0.950 ± 0.305 mg/L in rural Pakistan. In the Middle East, Homayonibezi et al. (2021) recorded the highest values, with 6.111 ± 0.501 mg/L in Asaluyeh and 9.135 ± 0.558 mg/L in Kaki.

##### 3.2.2.6 Manganese (Mn)

Manganese levels across the studies reviewed ranged from 0.04 ± 0.01 mg/L in Cyprus (Osório et al. 2015) to 0.75189 ± 0.31618 mg/L in Shandong, China (Guo et al. 2021) (Table 3). In Europe, Almášiová et al. (2023) measured 0.06 ± 0.05 mg/L in ecological Slovak farms and reported undetectable Mn in conventional farms, whereas Lauková et al. (2022) documented a mean of 0.0510 ± 0.0238 mg/L as the lowest among trace elements in Slovak goat milk. Miedico et al. (2016) found 0.0690 ± 0.0310 mg/L in Italian industrial regions. In East Asia, Zhou et al. (2017) observed 0.03378–0.04026 mg/L in rural Shaanxi and Shandong, whereas Chen et al. (2020) measured a mean of 0.156 ± 0.031 mg/L in the same provinces. Guo et al. (2021) recorded markedly higher concentrations, including 0.49546 ± 0.46136 mg/L in Shanxi, 0.57258 ± 0.35908 mg/L in Yunnan, and a peak of 0.75189 ± 0.31618 mg/L in Shandong. In the Middle East, Homayonibezi et al. (2021) found elevated levels of 0.239 ± 0.016 mg/L in Asaluyeh and 0.257 ± 0.027 mg/L in Kaki, Iran. In South Asia, Ahmad et al. (2017) reported 0.065 ± 0.032 mg/L in rural Pakistan.

### 3.3 Assessment of the Potential Health Risks Associated

#### 3.3.1 Estimated Daily Intake

The analysis of EDI values for toxic and potentially toxic elements in raw goat milk revealed consistently higher exposures among children compared to adults across all regions evaluated. This reflects body weight normalization and relative intake volume. Most studies reported values below the established tolerable daily intake (TDI) thresholds set by EFSA, WHO, or IOM; however, a wide range of EDI values was documented.

In Iran, Homayonibezi et al. (2021) reported the highest EDI values for chromium, with 5.87 × 10^−2^ mg/L bw/day in adults and 2.74 × 10^−1^ mg/L bw/day in children in Asaluyeh, and 5.08 × 10^−2^ and 2.37 × 10^−1^, respectively, in Kaki. Nickel, copper, iron, and manganese also showed high EDI values in these regions. In Pakistan's Karak District, Ahmad et al. (2017) reported elevated EDIs for zinc (1.19 × 10^−2^ adults; 5.58 × 10^−2^ children), chromium (4.11 × 10^−3^; 1.92 × 10^−2^), nickel (4.11 × 10^−3^; 1.92 × 10^−2^), and cadmium (2.64 × 10^−4^; 1.23 × 10^−3^).

Lebsir et al. (2023) documented moderate to high EDI values in Algeria, particularly in Djimla and El Milia. In Djimla, zinc reached 2.45 × 10^−2^ and cadmium 5.98 × 10^−4^ in children. Studies from China also reported relevant EDI values. Chen et al. (2020) observed Zn EDIs of 1.11 × 10^−2^ for adults and 5.18 × 10^−2^ for children; Zhou et al. (2017) reported 1.07 × 10^−2^ and 4.97 × 10^−2^, respectively. Guo et al. (2021) identified high Zn, Cu, and Mn EDIs in children from Yunnan, Shandong, and Shanxi.

In Slovakia, Lauková et al. (2022) presented EDI values for Zn, Cu, Fe, and Mn in both age groups, with children consistently showing higher intakes. Singh et al. (2019, 2014) reported elevated values for Fe (3.25 × 10^−2^) and Zn (1.82 × 10^−2^) in adults in India, with proportionally higher estimates for children. Lower EDI values were generally observed for mercury and arsenic. Kandhro et al. (2023) and Wanniatie et al. (2019) reported arsenic EDIs above 1.00 × 10^−3^ in children.

#### 3.3.2 Target Hazard Quotients

The THQ calculation for each metal provided a numerical estimate of non‐carcinogenic risk associated with chronic oral intake of raw goat milk. THQs were computed using EDI values derived from a standardized daily intake of 250 mL of milk, with separate body weight assumptions for adults (70 kg) and children (15 kg). A THQ value below 1 indicates exposure within the reference dose (RfD), whereas THQ ≥ 1 suggests potential risk, according to US EPA guidelines (US EPA 2009). Across studies, THQ values were consistently higher for children due to their lower body mass and proportionally greater intake (Table 4).

The highest THQ values were reported in Iran by Homayonibezi et al. (2021). In Asaluyeh, THQ‐Cr reached 19.6 for adults and 91.2 for children; in Kaki, values were 16.9 and 79.0, respectively. In Pakistan, Ahmad et al. (2017) found THQ‐Cd values of 2.64 for adults and 12.3 for children, and THQ‐Cr values of 1.37 and 6.40, respectively, in Karak District.

In Algeria, Lebsir et al. (2023) reported high THQ‐Cd values in El Milia (0.554 adults; 2.58 children), Texenna (0.142; 0.662), and Djimla (1.28; 5.98). In China, Chen et al. (2020) found THQ‐As values of 0.527 in adults and 2.46 in children, and additional values for Cr (0.0139; 0.0650), Ni, Cu, Zn, and Fe.

Other regions also showed relevant findings. In Eastern Europe, Korac et al. (2023) reported THQ‐Zn up to 0.244 and THQ‐Cu at 0.375 in children from Sarajevo. Singh et al. (2014) observed THQ‐Zn of 0.283 and THQ‐Fe of 0.217 in Indian children. Zhou et al. (2017), in China, reported THQ‐Zn of 0.166 in children, with lower values for Cu (0.0340), Al (0.00826), and As (0.00278). Miedico et al. (2016), in southern Italy, found low to moderate values for Cr (0.0117), Zn (0.194), and Fe (0.0202).

#### 3.3.3 Hazard Index

As with the EDI and THQ, HI values were consistently higher in children than adults, often by a factor of four to six (Table 5). Homayonibezi et al. (2021) reported the highest HI values, with 19.86 for adults and 92.68 for children in Asaluyeh, and 17.22 and 80.37, respectively, in Kaki, Iran. Gougoulias et al. (2014) observed HI values of 4.43 in adults and 20.67 in children in Thessaly, Greece. In Pakistan's Karak District, Ahmad et al. (2017) found HI values of 4.29 for adults and 19.99 for children.

**TABLE 4:** Estimated target hazard quotient (THQ) values for individual toxic and potentially toxic elements in raw goat milk.

Reference	Location	Zone	THQ (Adult/Child)
Ahmad et al. (2017)	Shnwa Gudi Khel, Distrito Karak, Pakistan	Rural zone	Cd: 2.64e + 00/1.23e + 01; Cr: 1.37e + 00/6.40e + 00; Ni: 2.06e − 01/9.60e − 01; Cu: 1.89e − 02/8.83e − 02; Zn: 3.98e − 02/1.86e − 01; Fe: 4.85e − 03/2.26e − 02; Mn: 1.66e − 03/7.74e − 03
Almášiová et al. (2023)	Orava, Slovakia	Rural zone	Cu: 1.07e − 02/5.00e − 02; Fe: 8.57e − 03/4.00e − 02; Mn: 1.53e − 03/7.14e − 03
	Stredné Považie, Slovakia	Rural zone	Zn: 2.80e − 02/1.31e − 01; Fe: 8.88e − 03/4.14e − 02
Chen et al. (2020)	Shandong and Shaanxi provinces, China	Rural zone	As: 5.27e − 01/2.46e + 00; Cd: 1.52e − 02/7.08e − 02; Cr: 1.39e − 02/6.50e − 02; Ni: 6.84e − 03/3.19e − 02; Cu: 1.86e − 02/8.67e − 02; Zn: 3.70e − 02/1.73e − 01; Fe: 5.51e − 03/2.57e − 02; Mn: 3.98e − 03/1.86e − 02
El‐Badry and Raslan (2016)	Zagazig city, Sharkia Governorate, Egypt	Rural zone	Cu: 7.68e − 05/3.58e − 04
Gougoulias et al. (2014)	Thessaly, Greece	Industrial zone	Cd: 4.29e + 00/2.00e + 01; Cr: 1.43e − 01/6.67e − 01
Guo et al. (2021)	Shandong, China	Rural zone	As: 9.06e − 02/4.23e − 01; Cr: 1.86e − 01/8.70e − 01; Cu: 7.72e − 02/3.60e − 01; Mn: 1.92e − 02/8.95e − 02
	Shanxi, China	Rural zone	As: 8.04e − 02/3.75e − 01; Cr: 3.88e − 02/1.81e − 01; Cu: 5.40e − 02/2.52e − 01; Mn: 1.26e − 02/5.90e − 02
	Yunnan, China	Rural zone	As: 1.30e − 01/6.08e − 01; Cr: 5.34e − 02/2.49e − 01; Cu: 5.92e − 02/2.76e − 01; Mn: 1.46e − 02/6.82e − 02
Homayonibezi et al. (2021)	Asaluyeh, Iran	Industrial zone	Cr: 1.96e + 01/9.12e + 01; Ni: 2.58e − 01/1.21e + 00; Cu: 1.30e − 02/6.08e − 02; Fe: 3.12e − 02/1.45e − 01; Mn: 6.10e − 03/2.85e − 02
	Kaki, Iran	Rural zone	Cr: 1.69e + 01/7.90e + 01; Ni: 2.36e − 01/1.10e + 00; Cu: 1.53e − 02/7.13e − 02; Fe: 4.66e − 02/2.17e − 01; Mn: 6.56e − 03/3.06e − 02
Ismail et al. (2015)	North West Multan, Pakistan	Residencial zone	Ni: 2.50e − 03/1.17e − 02; Cu: 2.18e − 02/1.02e − 01
	North east Multan, Pakistan	Residencial zone	Cd: 1.54e − 01/7.17e − 01; Cu: 3.04e − 02/1.42e − 01
	South east Multan, Pakistan	Residencial zone	Ni: 2.50e − 03/1.17e − 02; Cu: 4.04e − 02/1.89e − 01
	South west Multan, Pakistan	Industrial zone	Ni: 2.14e − 03/1.00e − 02; Cu: 6.19e − 02/2.89e − 01
Kandhro et al. (2023)	Hyderabad city, Pakistan	Rural zone	As: 1.48e + 00/6.89e + 00; Cd: 1.27e − 01/5.93e − 01
	Tharparkar, Pakistan	Rural zone	As: 3.87e − 01/1.81e + 00; Cd: 5.82e − 01/2.72e + 00
Korac et al. (2023)	Herzegovina	Rural zone	Cu: 1.87e − 02/8.75e − 02; Zn: 3.33e − 02/1.56e − 01
	Sarajevo, Bósnia and Herzegovina	Rural zone	Cu: 8.04e − 02/3.75e − 01; Zn: 5.24e − 02/2.44e − 01
Lauková et al. (2022)	Eastern Slovakia	Rural zone	Cu: 1.56e − 02/7.28e − 02; Zn: 3.05e − 02/1.42e − 01; Fe: 7.06e − 03/3.29e − 02; Mn: 1.30e − 03/6.07e − 03
Lebsir et al. (2023)	Djimla, Algeria	Rural zone	Cd: 1.28e + 00/5.98e + 00; Cu: 3.29e − 03/1.54e − 02; Zn: 1.75e − 02/8.15e − 02
	El Milia, Algeria	Industrial zone	Cd: 5.54e − 01/2.58e + 00; Cu: 4.46e − 03/2.08e − 02; Zn: 1.70e − 02/7.92e − 02
	Texenna, Algeria	Rural zone	Cd: 1.42e − 01/6.62e − 01; Cu: 5.65e − 03/2.64e − 02; Zn: 3.23e − 02/1.51e − 01
Miedico et al. (2016)	Manfredonia and Brindisi, Italy	Industrial zone	As: 6.07e − 02/2.83e − 01; Cd: 2.32e − 02/1.08e − 01; Al: 6.38e − 03/2.98e − 02; Cr: 2.50e − 03/1.17e − 02; Ni: 7.86e − 03/3.67e − 02; Cu: 6.88e − 03/3.21e − 02; Zn: 4.17e − 02/1.94e − 01; Fe: 4.34e − 03/2.02e − 02; Mn: 1.76e − 03/8.21e − 03
Osório et al. (2015)	Anogyra, Chipre	Rural zone	Zn: 2.11e − 02/9.83e − 02; Fe: 7.14e − 04/3.33e − 03; Mn: 2.30e − 03/1.07e − 02
	Cypirus, Chipre	Rural zone	Zn: 2.20e − 02/1.03e − 01; Fe: 1.63e − 03/7.62e − 03; Mn: 1.28e − 03/5.95e − 03
Osório et al. (2015)	Kofinou, Chipre	Rural zone	Al: 3.16e − 02/1.48e − 01; Zn: 1.48e − 02/6.89e − 02; Fe: 8.16e − 04/3.81e − 03; Mn: 1.02e − 03/4.76e − 03
Parsaei et al. (2019)	Isfahan Province, Iran	Rural zone	Hg: 1.82e − 01/8.50e − 01; Cd: 1.15e − 01/5.37e − 01
Singh et al. (2014)	Hisar, India	Rural zone	Zn: 6.07e − 02/2.83e − 01; Fe: 4.64e − 02/2.17e − 01
Singh et al. (2019)	Karnal, India	Rural zone	Cu: 4.46e − 02/2.08e − 01; Zn: 5.71e − 02/2.67e − 01; Fe: 3.98e − 03/1.86e − 02; Mn: 7.65e − 03/3.57e − 02
Wanniatie et al. (2019)	Bagor, Indonesia	Rural zone	As: 8.33e − 01/3.89e + 00; As: 1.31e + 00/6.11e + 00
Zhou et al. (2017)	Shaanxi and Shandong, China	Rural zone	Hg: 3.93e − 03/1.83e − 02; As: 5.95e − 04/2.78e − 03; Cd: 0.00e + 00/0.00e + 00; Al: 1.77e − 03/8.26e − 03; Cr: 4.88e − 04/2.28e − 03; Ni: 3.13e − 04/1.46e − 03; Cu: 7.28e − 03/3.40e − 02; Zn: 3.55e − 02/1.66e − 01; Fe: 1.84e − 03/8.60e − 03; Mn: 8.62e − 04/4.02e − 03

*Note*: THQ values are presented separately for adults and children and are based on chronic daily intake. Values greater than 1 indicate potential non‐carcinogenic health risks. Only studies that provided complete and quantifiable data are included. Elemental abbreviations follow IUPAC standards; values are expressed in scientific notation (Adult/Child).

Unexpectedly high HI values were also documented in Eastern Slovakia. Lauková et al. (2022) reported values of 7.10 in adults and 33.14 in children. In Indonesia, Wanniatie et al. (2019) recorded adult HI values of 1.78 and 2.86 and child HI values of 8.33 and 13.33 in conventional and organic farms, respectively.

Other studies reported moderate HI values in adults but higher values in children. Guo et al. (2021) observed adult HI values below 0.38 and up to 1.74 in children across three Chinese provinces. Parsaei et al. (2019) documented HI values of 0.30 in adults and 1.39 in children in Iran. In Algeria, Lebsir et al. (2023) reported HI values ranging from 0.18 to 1.30 for adults and 0.83 to 6.08 for children across different sites.

In contrast, Almášiová et al. (2023) reported the lowest HI values, with adult values between 0.02 and 0.04 and child values between 0.09 and 0.17 in Slovakia. Similarly low values were observed by Osório et al. (2015) in Cyprus and Singh et al. (2015) in India, where adult HI remained below 0.13 and child HI below 0.61. In China, Chen et al. (2020) reported a moderate HI of 0.628 for adults and 2.93 for children.

#### 3.3.4 Carcinogenic Risk Assessment

The CRA based on arsenic (As) and chromium (Cr) exposure in raw goat milk demonstrated notable variation across geographic regions, environmental conditions, and age groups. CRA values were calculated by multiplying the EDI by the corresponding slope factor (SF) and compared to the United States EPA's benchmark range for acceptable carcinogenic risk (1 × 10^−6^ to 1 × 10^−4^).

For adults, most CRA values fell within the acceptable range. In children, however, values often exceeded this threshold by one or two orders of magnitude, reflecting their higher intake per unit of body weight. Among the highest values were those reported by Homayonibezi et al. (2021): CRA‐Cr reached 2.93 × 10^−2^ in adults and 1.37 × 10^−1^ in children in Asaluyeh and 2.54 × 10^−2^ and 1.18 × 10^−1^, respectively, in Kaki.

In Pakistan, Ahmad et al. (2017) found CRA‐Cr values of 2.06 × 10^−3^ in adults and 9.60 × 10^−3^ in children in Karak District. Zhou et al. (2017) and Guo et al. (2021) also reported elevated pediatric CRA‐Cr values in rural China. Zhou documented 3.42 × 10^−3^ in children, and Guo reported values ranging from 2.72 × 10^−4^ to 1.31 × 10^−3^.

CRA‐As values followed similar regional patterns. Kandhro et al. (2023) recorded 6.64 × 10^−4^ in adults and 3.10 × 10^−3^ in children in Hyderabad and 1.74 × 10^−4^ and 8.12 × 10^−4^, respectively, in Tharparkar. Chen et al. (2020) found CRA‐As at 2.37 × 10^−4^ in adults and 1.11 × 10^−3^ in children. Additional pediatric values above 1 × 10^−3^ were reported by Guo et al. (2021) and Zhou et al. (2017). Lower CRA values were documented by Miedico et al. (2016) in southern Italy (CRA‐As: 2.73 × 10^−5^ in adults, 1.28 × 10^−4^ in children) and by Gougoulias et al. (2014) in Greece (CRA‐Cr: 2.14 × 10^−4^ in adults, 1.00 × 10^−3^ in children).

## 4 Discussion

### 4.1 Toxic and Potentially Toxic Metals

The distribution of research focus across elements within the compiled dataset reflects regulatory attention and the convergence of toxicological significance and environmental prevalence. Arsenic, lead, cadmium, chromium, nickel, zinc, copper, and iron emerged as the most frequently quantified elements, underscoring their dual status as regulated toxicants and essential micronutrients with narrow physiological thresholds. By contrast, despite their well‐documented neurotoxic and systemic hazards, mercury, aluminum, and manganese were investigated in only two to five studies, exposing critical surveillance gaps likely contributing to underestimating their role in cumulative dietary exposure. This imbalance is compounded by two overarching limitations that permeate the current evidence base: the near‐universal absence of metal speciation, particularly for chromium (Cr(III) vs. Cr(VI)) and mercury (inorganic vs. methylated forms), and the pronounced heterogeneity of reported concentrations across studies and regions. Without chemical speciation, risk estimates are necessarily conservative, often defaulting to worst‐case assumptions, such as treating all detected chromium as Cr(VI), a Group 1 IARC carcinogen, which both inflates toxicity estimates and underscores the urgent need for refined analytical resolution in future studies (IARC 2012).

Within this landscape, lead and cadmium consistently registered as critical concerns, with concentrations surpassing the European limit of 0.02 mg/L in multiple high‐risk settings. Elevated Pb burdens, such as those reported in Djimla, Algeria (0.382 ± 0.0026 mg/L), and Asaluyeh, Iran (0.141 ± 0.030 mg/L), together with Cd maxima in Greece (0.12 ± 0.02 mg/L) and rural Pakistan (0.074 ± 0.003 mg/L), highlight contamination pathways that extend beyond industrial epicenters. The convergence of atmospheric deposition, phosphate fertilizer application, and wastewater irrigation drives soil contamination, which propagates into forages and milk, as evidenced by quantified soil‐to‐pasture and pasture‐to‐milk transfer factors (Castro et al. 2024). These diffuse mechanisms challenge the assumption that rural systems inherently offer protection from heavy metal exposure, as several agricultural zones, including Djimla, Texenna, and Tharparkar, rival or exceed industrial regions in Pb burdens, complicating geographic risk profiling (Table 2).

Although less frequently monitored, mercury's occurrence underscores the importance of dietary and feed‐based vectors in shaping milk burdens (Almeida et al. 2022). Detectable levels in Iran (0.00510 ± 0.00050 mg/L) and its correlation with feed inputs in China reinforce the need to scrutinize imported concentrates and additive use as vehicles of exposure, especially where regional controls are limited (Guo et al. 2021; Zohra et al. 2022). Arsenic, more broadly represented than Hg or Al, exhibited extreme variability, ranging from undetectable levels (Almášiová et al. 2024) to concentrations exceeding Codex Alimentarius thresholds, as observed in Hyderabad (0.124 ± 0.00082 mg/L) and Djimla (0.11 ± 0.00019 mg/L). Its persistence across both organic and conventional production systems underscores the primacy of soil geochemistry, aquifer contamination, and atmospheric inputs as dominant vectors, rather than farming practices per se (Patel et al. 2023).

Chromium stands out for its divergence in reported concentrations among potentially toxic yet essential elements. Although most studies documented Cr levels below 0.2 mg/L, industrial zones such as Asaluyeh and Kaki reported values exceeding 14 mg/L (Homayonibezi et al. 2021), magnitudes atypical for dairy matrices. Although these findings align with these regions’ petrochemical and alkaline soil context, their scale relative to the broader dataset signals the need for heightened analytical validation, including unit verification and cross‐method comparison, before integrating into global exposure models. Similarly, nickel, reported by nine studies, was markedly elevated in Asaluyeh (1.447 ± 0.101 mg/L) and rural districts in Pakistan (>1 mg/L), reflecting its high ionic mobility in saline and semi‐arid soils subject to industrial effluent loads (Fechete et al. 2024). The co‐occurrence of Ni and Cr in petrochemical belts raises concerns about synergistic oxidative stress, a mechanism insufficiently addressed in current risk frameworks and deserving of targeted toxicological exploration.

Essential elements zinc and copper, each present in over 13 studies, highlight a different axis of concern. Although Zn exceeded 5 mg/L in India and Sarajevo, and Cu reached 0.86476 ± 0.49038 mg/L in Shandong, such concentrations, though not overtly toxic, risk perturbing mineral homeostasis and potentiating hepatic and immune stress, particularly in concert with elevated Fe and Mn (Shkembi and Huppertz 2021). Iron displayed significant variability, with concentrations exceeding 9 mg/L in Kaki and Hisar, which may contribute to overload in sensitive populations and indicate shared contamination pathways with other transition metals (Fatoki and Badmus 2022). Aluminum, despite detection in five studies, including a peak of 1.24 ± 0.35 mg/L in Cyprus (Table 2), remains under‐characterized relative to its neurotoxic potential, warranting its inclusion in future monitoring frameworks (Ofoe et al. 2023).

Taken together, these patterns delineate three central insights. First, industrial centers, exemplified by Asaluyeh, represent hotspots for extreme Cr and Ni accumulation, demanding intensified monitoring and regionally adaptive risk thresholds. Second, rural and ostensibly low‐risk areas, notably in Algeria and Pakistan, can equal or surpass urban centers in Pb and Cd loads due to diffuse anthropogenic drivers, challenging conventional assumptions about geographic exposure gradients. Third, Europe, particularly Slovakia, serves as a benchmark for regulatory efficacy, with most studies reporting <LOD for targeted metals. These findings, although valuable as a global comparative baseline, also illuminate critical gaps, including the absence of speciation data, the paucity of harmonized methodologies, and the near‐total lack of coverage for Latin America and Sub‐Saharan Africa, which collectively limit the robustness and generalizability of current risk appraisals.

### 4.2 Risk of Assessment

Building on the pronounced elemental and geographic disparities highlighted previously, where Pb and Cd frequently surpassed regulatory limits across both rural and industrial landscapes and chromium displayed extreme outliers in petrochemical regions, the integrated assessment of risk through EDI, THQ, HI, and CRA collectively reveals a toxicological panorama that is far more complex and spatially heterogeneous than what single‐element concentration data alone would suggest. Although raw goat milk is often valorized as a health‐promoting alternative to bovine milk, particularly in marginal or agroecologically vulnerable regions, this review demonstrates that it may also act as a consistent vector for chronic exposure to toxic and potentially toxic elements, including those classified as carcinogens. The convergence of findings across models reinforces the hypothesis that environmental and dietary exposure through goat milk constitutes a low‐visibility but biologically significant contributor to long‐term health risks, especially in children. This concern regarding the vulnerability of pediatric populations to environmental contaminants is broadly corroborated by systematic reviews of other chemical classes, such as phthalate esters in drinking water sources, which similarly highlight a substantial proportion of children at risk on the basis of estimated exposures (Vasseghian et al. 2023, 2024). Across the dataset, EDI values revealed a consistent trend of elevated exposure in pediatric populations, driven by lower body mass and higher intake per kilogram of weight.

EDI calculations consistently underscore the elevated vulnerability of children, whose higher milk intake relative to body weight amplifies their exposure burden. Although adult EDI values largely remained within TDI thresholds, pediatric estimates frequently approached or exceeded conservative benchmarks, particularly in regions where goat milk is a dietary cornerstone. This elevation extended beyond classic toxicants. As shown in Table 3, essential elements such as zinc (Zn), copper (Cu), and iron (Fe) also reached levels capable of eliciting oxidative stress, hepatic overload, and disruption of trace element homeostasis. For example, Zn EDI values in children reached 5.58 × 10^−2^ mg/L bw/day in Shnwa Gudi Khel, Pakistan (Ahmad et al. 2017), and 8.50 × 10^−2^ mg/L bw/day in Hisar, India (Singh et al. 2014). For Cu, pediatric EDI values peaked at 1.44 × 10^−2^ mg/L bw/day in Shandong, China (Guo et al. 2021) and 1.15 × 10^−2^ mg/L bw/day in Multan, Pakistan (Ismail et al. 2015). Iron burdens were even more striking, with EDI values of 1.52 × 10^−1^ mg/L bw/day recorded in Kaki, Iran (Homayonibezi et al. 2021) and Hisar, India (Singh et al. 2014). This dual exposure, where toxic metals and essential nutrients simultaneously exceed safe thresholds, highlights the limitations of relying solely on elemental concentrations for public health risk evaluation, as nutrient overload can potentiate or modulate the toxicodynamics of other metals.

THQ modeling refines this picture by quantifying non‐carcinogenic risk for individual elements. Across numerous studies, THQ values for Cr, Cd, and As exceeded the critical threshold of 1, often by one or more orders of magnitude in children (Ahmad et al. 2017; Homayonibezi et al. 2021; Lebsir et al. 2023). Zn and Cu, typically considered benign, also yielded elevated THQs in some regions (Singh et al. 2014; Korac et al. 2023), reinforcing that cumulative nutrient burdens can act as toxicological stressors when exposures surpass homeostatic thresholds.

The HI broadens the lens by integrating the cumulative burden of multiple elements, revealing that multi‐metal exposure through goat milk is neither theoretical nor rare but actively occurring across diverse environmental and socio‐economic contexts. As shown inTable 5, children in petrochemical regions of Iran bore the most extreme risks, with Homayonibezi et al. (2021) reporting HI values of 92.7 in Asaluyeh and 80.4 in Kaki. Even outside such industrial contexts, HI values far exceeded the threshold of concern: Gougoulias et al. (2014) documented an HI of 20.67 among children in Thessaly, Greece; Ahmad et al. (2017) reported an HI of 20.0 in Karak, Pakistan; and Lauková et al. (2022) found values of 0.254 in Eastern Slovakia. In mining‐impacted Pakistan, Kandhro et al. (2023) reported pediatric HIs of 7.48 in Hyderabad and 4.52 in Tharparkar, whereas Wanniatie et al. (2019) observed HIs of 3.89 and 6.11 in children from conventional and organic farms, respectively, in Indonesia. These patterns corroborate that multi‐element exposure is pervasive and not restricted to industrial zones alone.

However, interpreting HI results demands caution due to its inherent structural limitations. By summing THQs linearly, HI assumes strictly additive effects, disregarding synergistic, antagonistic, and potentiating interactions between metals. These processes are increasingly recognized as central to the toxicological behavior of low‐dose, multi‐agent mixtures (EPA 2001). For instance, simultaneous exposure to Fe, Zn, and Cu, though each element falls below its respective safety threshold, can perturb mineral homeostasis and amplify oxidative stress through redox cycling, outcomes invisible to the HI framework. Overreliance on HI as a solitary indicator risks underestimating or mischaracterizing real‐world health hazards. Future risk assessment must move beyond linear models toward frameworks incorporating interaction‐weighted indices, dynamic dose–response relationships, and probabilistic tools such as Monte Carlo simulations or Bayesian networks (Wang et al. 2023). Such models, underpinned by robust chemical speciation data, would yield more accurate and biologically relevant risk characterizations.

By addressing lifetime carcinogenic risk, the CRA model adds a final, critical dimension to this assessment. CRA values for As and Cr in children frequently exceeded 1 × 10^−3^ and, in several instances, approached 1 × 10^−1^, far surpassing the United States EPA's upper risk benchmark of 1 × 10^−4^. The highest values were again documented in petrochemical Iran, with Homayonibezi et al. (2021) reporting CRA‐Cr values of 1.37 × 10^−1^ in Asaluyeh and 1.18 × 10^−1^ in Kaki (Table 6). Elevated CRA‐Cr values were also reported in less industrialized contexts, including 9.60 × 10^−3^ in Karak, Pakistan (Ahmad et al. 2017), 3.42 × 10^−3^ in rural China (Zhou et al. 2017), and up to 1.31 × 10^−3^ in Shandong (Guo et al. 2021). CRA‐As patterns mirrored these regional disparities, with Kandhro et al. (2023) documenting 3.10 × 10^−3^ in Hyderabad and 8.12 × 10^−4^ in Tharparkar, Pakistan, whereas Chen et al. (2020) reported 1.11 × 10^−3^ in China, and additional values above 1 × 10^−3^ were noted by Zhou et al. (2017) and Guo et al. (2021). These magnitudes, though alarming, must be contextualized by the absence of speciation data across nearly all studies, which necessitated conservative assumptions, treating all detected Cr as Cr(VI), a Group 1 IARC carcinogen (IARC 2012), that may inflate estimates. Although such assumptions are defensible in petrochemical belts and aquifers prone to hexavalent chromium, they contribute to uncertainty in meta‐analytical syntheses. Furthermore, the chromium concentrations underpinning these CRA estimates, particularly those reported by Homayonibezi et al. (2021), deviate starkly from the broader dataset (where Cr rarely exceeded 0.2 mg/L). Although Asaluyeh's industrial profile may partially justify elevated values, these outliers warrant critical scrutiny for possible methodological inconsistencies, including unit misreporting, contamination, or uncorrected dilution, and should undergo independent validation before integration into global risk baselines.

**TABLE 5:** Hazard index (HI) values for cumulative non‐carcinogenic risk from metal exposure through raw goat milk consumption.

Reference	Location	Zone	HI adult.	HI child.
Zhou et al. (2017)	Shaanxi and Shandong, China	Rural zone	5.26e − 02	2.45e − 01
Lauková et al. (2022)	Eastern Slovakia	Rural zone	5.44e − 02	2.54e − 01
Singh et al. (2014)	Hisar, India	Rural zone	1.07e − 01	5.00e − 01
Korac et al. (2023)	Sarajevo, Bósnia and Herzegovina	Rural zone	1.33e − 01	6.19e − 01
	Herzegovina	Rural zone	5.21e − 02	2.43e − 01
Kandhro et al. (2023)	Hyderabad city	Rural zone	1.60e + 00	7.48e + 00
	Tharparkar Pakistan	Rural zone	9.69e − 01	4.52e + 00
Homayonibezi et al. (2021)	Asaluyeh	Industrial zone	1.99e + 01	9.27e + 01
	Kaki	Rural zone	1.72e + 01	8.04e + 01
Lebsir et al. (2023)	El Milia	Industrial zone	5.75e − 01	2.68e + 00
	Texenna	Rural zone	1.80e − 01	8.39e − 01
	Djimla	Rural zone	1.30e + 00	6.08e + 00
Ismail et al. (2015)	South west Multan, Pakistan	Industrial zone	6.40e − 02	2.99e − 01
	North West Multan, Pakistan	Residencial zone	2.43e − 02	1.13e − 01
	South east Multan, Pakistan	Residencial zone	4.29e − 02	2.00e − 01
	North east Multan, Pakistan	Residencial zone	1.84e − 01	8.58e − 01
Guo et al. (2021)	Yunnan, China	Rural zone	2.57e − 01	1.20e + 00
	Shandong, China	Rural zone	3.73e − 01	1.74e + 00
	Shanxi, China	Rural zone	1.86e − 01	8.67e − 01
Miedico et al. (2016)	Manfredonia and Brindisi, Italy	Industrial zone	1.55e − 01	7.25e − 01
Parsaei et al. (2019)	Isfahan Province, Iran	Rural zone	2.97e − 01	1.39e + 00
Ahmad et al. (2017)	Shnwa Gudi Khel, Distrito Karak	Rural zone	4.29e + 00	2.00e + 01
Singh et al. (2019)	Karnal, India	Rural zone	1.13e − 01	5.29e − 01
Chen et al. (2020)	Shandong and Shaanxi provinces, China	Rural zone	6.28e − 01	2.93e + 00
Almášiová et al. (2023)	Orava	Rural zone	2.08e − 02	9.71e − 02
	Stredné Považie	Rural zone	3.69e − 02	1.72e − 01
El‐Badry and Raslan (2016)	Zagazig city, Sharkia governorate, Egypt	Rural zone	7.68e − 05	3.58e − 04
Gougoulias et al. (2014)	Grécia, Thessaly	Industrial zone	4.43e + 00	2.07e + 01
Wanniatie et al. (2019)	Bagor, Indonesia	Rural zone	8.33e − 01	3.89e + 00
	Bagor, Indonesia	Rural zone	1.31e + 00	6.11e + 00
Osório et al. (2015)	Anogyra, Chipre	Rural zone	2.41e − 02	1.12e − 01
	Kofinou, Chipre	Rural zone	4.82e − 02	2.25e − 01
	Cypirus, Chipre	Rural zone	2.49e − 02	1.16e − 01
Bilandžić et al. (2015)	Croatia	Rural zone	1.27e − 01	5.94e − 01

*Note*: HI values represent the sum of individual target hazard quotients (THQs) for each element detected in a given study. Results are presented separately for adults and children. An HI ≥ 1 indicates a potential non‐carcinogenic health risk due to the additive effects of multiple metals. Only studies with available quantitative data for at least two elements are included. Elemental contributions were weighted on the basis of their respective reference doses (RfD).

**TABLE 6:** Carcinogenic risk (CRA) estimates for arsenic (As) and chromium (Cr) exposure through raw goat milk consumption.

References	Location	Zone	CRA As (Adult)	CRA As (Child)	CRA Cr (Adult)	CRA Cr (Child)
Zhou et al. (2017)	Shaanxi and Shandong, China	Rural	2.68e − 07	1.25e − 06	7.32e − 04	3.42e − 03
Kandhro et al. (2023)	Hyderabad city	Rural zone	6.64e − 04	3.10e − 03		
	Tharparkar Pakistan	Rural zone	1.74e − 04	8.12e − 04		
Homayonibezi et al. (2021)	Asaluyeh	Industrial			2.93e − 02	1.37e − 01
	Kaki	Rural			2.54e − 02	1.18e − 01
Guo et al. (2021)	Yunnan, China	Rural	5.86e − 05	2.74e − 04	8.01e − 05	3.74e − 04
	Shandong, China	Rural	4.08e − 05	1.90e − 04	2.80e − 04	1.31e − 03
	Shanxi, China	Rural	3.62e − 05	1.69e − 04	5.82e − 05	2.72e − 04
Miedico et al. (2016)	Manfredonia and Brindisi, Italy	Industrial	2.73e − 05	1.28e − 04	3.75e − 06	1.75e − 05
Ahmad et al. (2017)	Shnwa Gudi Khel, Karak	Rural			2.06e − 03	9.60e − 03
Bilandžić et al. (2015)	Croatia	Rural			1.29e − 04	6.00e − 04
Chen et al. (2020)	Shandong and Shaanxi, China	Rural	2.37e − 04	1.11e − 03	2.09e − 05	9.75e − 05
Gougoulias et al. (2014)	Thessaly, Greece	Rural			2.14e − 04	1.00e − 03

*Note*: Values are expressed for adult and child populations, based on individual slope factors (SF) established by the United States EPA. The total CRA represents the cumulative cancer risk from simultaneous exposure to As and Cr. According to regulatory standards, CRA values below 1 × 10^−6^ are considered negligible, values between 1 × 10^−6^ and 1 × 10^−4^ fall within the acceptable risk range, and values above 1 × 10^−4^ indicate a potential lifetime carcinogenic concern.

Viewed collectively, the outputs of EDI, THQ, HI, and CRA construct a coherent, if sobering, narrative: raw goat milk is not intrinsically hazardous, yet its safety is profoundly context‐dependent, shaped by environmental burdens, production practices, and the capacity of local food safety systems. The same product that delivers high‐quality nutrients in Slovakia (Almášiová et al. 2023) or Cyprus (Osório et al. 2015) can simultaneously act as a vector for chronic multi‐metal exposure in Pakistan, Iran, or China, not as isolated incidents of contamination, but as systemic reflections of agroecological and socio‐environmental pressures.

## 5 Conclusion

This systematic review demonstrates that raw goat milk, while nutritionally vital and culturally entrenched across diverse regions, also serves as a conduit for chronic exposure to toxic and potentially toxic metals, with risks shaped as much by environmental context and production practices as by the elemental profiles themselves. Lead and cadmium emerged as persistent hazards in both rural and industrial systems, chromium and nickel showed extreme accumulations in petrochemical landscapes, and even essential elements such as zinc, copper, and iron frequently reached concentrations capable of disrupting physiological balance. These burdens translated into consistently elevated risk metrics, with children bearing the greatest vulnerability, and revealed that goat milk's role extends beyond nutrition to that of a sentinel bioindicator of diffuse environmental contamination. Addressing these intertwined benefits and risks demands a scientific and regulatory shift toward harmonized, regionally adaptive monitoring frameworks that integrate chemical speciation, interaction‐based and probabilistic risk models, and source tracking. Only through such integrative approaches can goat milk's dual role, as a dietary resource and a diagnostic matrix, be leveraged to protect public health and inform context‐sensitive, evidence‐driven policy.

## Author Contributions

**Arlen Carvalho de Oliveira Almeida**: conceptualization, investigation, writing – original draft, methodology, writing – review and editing, data curation. **Paloma Almeida Rodrigues**: conceptualization, investigation, writing – review and editing. **Marion Pereira da Costa**: conceptualization, investigation, writing – review and editing. **Carlos Adam Conte‐Junior**: conceptualization, investigation, funding acquisition, resources, writing – review and editing, supervision.

## Ethics Statement

The authors have nothing to report.

## Conflicts of Interest

The authors declare no conflicts of interest.

## Data Availability

The datasets generated and analyzed during the current study are available from the corresponding author on reasonable request.
